# Angiogenin Promotes U87MG Cell Proliferation by Activating NF-κB Signaling Pathway and Downregulating Its Binding Partner FHL3

**DOI:** 10.1371/journal.pone.0116983

**Published:** 2015-02-06

**Authors:** Wenrong Xia, Wenliang Fu, Xin Cai, Min Wang, Huihua Chen, Weiwei Xing, Yuanyuan Wang, Minji Zou, Tao Xu, Donggang Xu

**Affiliations:** Laboratory of Genome Engineering, Beijing Institute of Basic Medical Sciences, Beijing, PR China; Wayne State University School of Medicine. UNITED STATES

## Abstract

Angiogenin (Ang) is known to induce cell proliferation and inhibit apoptosis by cellular signaling pathways and its direct nuclear functions, but the mechanism of action for Ang in astrocytoma is not yet clear. Astrocytoma is the most frequent one among various neurogliomas, of which a subtype known as glioblastoma multiforme (GBM) is the most malignant brain glioma and seriously influences the life quality of the patients. The expression of Ang and Bcl-xL were detected in 28 cases of various grades of astrocytoma and 6 cases of normal human tissues by quantitative real-time PCR. The results showed that the expression of Ang and Bcl-xL positively correlated with the malignant grades. Cytological experiments indicated that Ang facilitated human glioblastoma U87MG cell proliferation and knock-down of endogenous Ang promoted cell apoptosis. Furthermore, Ang activated NF-κB pathway and entered the U87MG cell nuclei, and blocking NF-κB pathway or inhibiting Ang nuclear translocation partially suppressed Ang-induced cell proliferation. The results suggested that Ang participated in the regulation of evolution process of astrocytoma by interfering NF-κB pathway and its nucleus function. In addition, four and a half LIM domains 3 (FHL3), a novel Ang binding partner, was required for Ang-mediated HeLa cell proliferation in our previous study. We also found that knockdown of FHL3 enhanced IκBα phosphorylation and overexpression of Ang inhibited FHL3 expression in U87MG cells. Together our findings suggested that Ang could activate NF-κB pathway by regulating the expression of FHL3. In conclusion, the present study established a link between Ang and FHL3 proteins and identifies a new pathway for regulating astrocytoma progression.

## Introduction

Angiogenin (Ang) was initially isolated from serum-free supernatants of an established human adenocarcinoma cell line (HT-29) [1], but it was not a tumor-specific product. The expression of Ang was shown to be up-regulated in numerous tumors [2], which was also found in normal cells and human plasma [3]. Ang is the first known human tumor-derived protein with *in vivo* angiogenic activity, but may also have some other biological activities in addition to angiogenesis. Ang protected cultured motoneurons against excitotoxic injury in a PI-3-kinase/Akt kinase-dependent manner, whereas knock-down of Ang potentiated excitotoxic motoneuron death [4]. Ang also activated ERK1/2 and B/Akt in human umbilical vein endothelial cells and induced phosphorylation of SAPK/JNK in human umbilical artery smooth muscle cells [5–6]. It also inhibited serum withdrawal-induced apoptosis by activating NF-κb-mediated cellular survival pathway and Bcl-2-mediated anti-apoptotic pathway in pluripotent P19 mouse embryonal carcinoma cells [7–8]. Furthermore, Ang bound to the promoter region of rDNA and stimulate rRNA transcription, so direct nuclear function of Ang was required for Ang-induced cell proliferation [9]. Aminoglycoside antibiotics neomycin and neamine have been shown to block nuclear translocation of ANG thereby abolishing the biological activity of ANG and inhibiting cancer cell proliferation as well as tumor angiogenesis [10]. ANG also mediated androgen-independent rRNA transcription and underwent constitutive nuclear translocation in androgen-insensitive PCa cells, resulting in a constant rRNA overproduction thereby stimulating cell proliferation [11].

Brain astrocytoma is the most frequent one among the various neurogliomas, the glioblastoma multiforme (GBM) out of which is the most malignant brain glioma subtype. Although there have been treatment methods at present, the prognosis is very poor and the life quality of patients was seriously influenced [12]. In the process of genesis, development and malignant transformation, the expression of different signaling molecules all can accelerate or delay the progress of patients’ condition. NF-κB pathway is considered as one of the treatment targets and in the activated state in GBM, blocking of which facilitated senescence of the differentiated cells [13]. Ang is detectable in different kinds of intracranial tumors with the lowest amount in low-grade astrocytomas and contributes to the malignant transformation of gliomas [14]. To further elucidate the molecular mechanisms by which Ang regulates tumor growth and progression, we detected the expression of Ang in different grade of astrocytoma and whether Ang can promote U87MG cell proliferation via NF-κB pathway and its nucleus function.

Furthermore, the research for Ang is not yet completed and its mechanism of action is still unclear. We started from the interaction proteins of Ang to explore the possible mechanism of Ang in cell proliferation. Four and a half LIM domains 3 (FHL3), a member of the LIM family, was identified as a novel Ang binding partner in our previous study [15]. The results showed that direct interaction between Ang and FHL3 exists both *in vivo* and *in vitro*. Moreover, FHL3 is required for Ang-mediated HeLa cell proliferation by influencing nuclear translocation of Ang. In our study, we found that FHL3 may be involved in Ang-activated NF-κB pathway.

## Materials and Methods

### Patients and tissue samples

All the tissue samples were collected from the operative inpatients in the Department of Neurosurgery, the Second Hospital of Hebei Medical University, that was approved by Local and Medical Ethics Committee (Second Hospital of Hebei Medical Research Ethics Committee, Approval No. 2012003). We confirmed that the written informed consent was obtained from the next of kin in this study and the data of samples were analyzed anonymously. Our clinical investigation has been conducted according to the principles formulated by the Declaration of Helsinki. Among the 34 samples, 28 cases were diagnosed as astrocytoma and graded according to WHO’s Histopathological Grading (2000A.D.): 8 grade Ⅱ of 8 cases, grade Ⅲ of 11 cases, grade Ⅳ of 9 cases. In addition, 6 normal control samples were the tissues resected from the patients with traumatic brain injury treated by the brain decompression. Meanwhile, all the pathological results were diagnosed by two senior physicians from the Department of Pathology. A part of the fresh tissue samples was stored in liquid nitrogen, and used for analysis of the expression levels of Ang, Bcl-xL and FHL3 by at mRNA or protein levels. The primer sequences for RT-qPCR were as follows:

Ang-qf: 5’-ACACTTCCTGACCCAGCACT-3’;
Ang-qr: 5’-TGTTTTCACAGATGGCCTTG-3’;
Bcl-xL-qf: 5’-GAGGCAGGCGACGAGTTTGAAC-3’;
Bcl-xL-qr: 5’-GCTGCGATCCGACTCACCAATAC-3’;
FHL3-qf: 5’-GTCCCTGTATGGACGCAAGT-3’;
FHL3-qr: 5’-GAAGGGTTCATCGGCTAGTG-3’;
GAPDH-qf: 5’-GAGTCAACGGATTTGGTCGT-3’;
GAPDH-qr: 5’-TTGATTTTGGAGGGATCTCG-3’

### Cell lines

Human glioblastoma U87MG cells (The Cell Center of Union Medical College, Beijing, China) were maintained in RPMI-1640 medium (Hyclone, Logan, Utah, USA) supplemented with 10% fetal bovine serum (Gibco, Grand Island, NY, USA), at 37°C with 5% CO2.

### MTT assay

Various concentrations of rhAng (R&D, Minneapolis, MN, USA) or rhFHL3 (GeneTex, Irvine, CA, USA) was added to 96-well plates containing 5,000 U87MG cells/well. After incubation for 48 h, cell growth was measured by MTT (3-(4, 5-dimethylthiazolyl-2)-2,-diphenyltetrazoliumbromide) proliferation assay. Absorbance at 490 nm was measured using a Bio-Rad model 550 microplate reader (BioRad Molecular Bioscience Group, Hercules, USA). The data represent the means of three independent experiments.

### Plasmids, siRNAs, and transfection

The cDNA target sequences of siRNAs for Ang were CCAGCACUAUGAUGCCAAATT which was cloned into pGPU6/GFP/Neo-shRNA （shAng）. The pCMV-myc/Ang expression construct has been described previously [15]. The cDNA target sequence of siRNAs for FHL3 was AAGTACATCCAGACAGACAGC. All of the plasmids were purified using the Plasmid Mini kit (Omega, Norcross, GA, USA). Transfections were performed using Lipofectamine 2000 (Invitrogen, Grand Island, NY, USA).

### Measurement of Apoptosis

Double staining method with AnnexinV-FITC/Propidium iodide (PI) (Nanjing Keygen Biotech. Co, Ltd, China) was used to detect the phosphatidylserine(PS) at the cell surface to calculate the apoptotic cell number. U87MG cells were inoculated at 6×10^5^/well into a 6-well plate, which were divided into negative control and shAng-transfected group. The cells were collected after transfection with Lipofectamine 2000 for 48h, digested with 0.25% trypsin without EDTA (Invitrogen, USA) and washed with PBS for 2 times. After cells were resuspended in 500μl binding buffer, 5μl Annexin V-FITC and 5μl PI were added and mixed homogeneously, then stained in dark for 15min. The stained cells were counted and analyzed by flow cytometry (FACSCalibur, BD Bioscience).

### Preparation of protein lysates

U87MG cells were cultured for 24h, washed with RPMI-1640 medium for three times and serum-starved for another 12h. The cells were then incubated with different concentration of rhAng for 2h. Nuclear proteins were extracted by Nuclear Extraction Kit (CWbiotech, Beijing, China) and total proteins were extracted using RIPA lysis buffer. Samples with equal amounts of protein were subject to SDS-PAGE and Western blotting, so as to analyse the phosphorylation of IκBα (Epitomics, Burlingame, CA, USA), p65 (Epitomics), Bcl-xL (Cell Signaling Technology, Boston, USA), Ang (R&D Systems), Histone H3 (Beyotime institute of biotechnology, Haimen, JS, China) and GAPDH (CWBiotech).

### Inhibition of NF-κB signaling pathway

U87MG cells were inoculated in a 6-well plate at 80% cell density, and then the medium was changed to serum-free medium for starvation for 12h. The cells were pretreated by IKK inhibitor (EMD chemicals, Gibbstown, NJ, USA) for 1h [16], and then were stimulated by Ang at the final concentration 1μg/ml for 2h followed by protein extraction. U87MG cells were seeded in a 96-well plate at 5,000 cells/well for 24h, and then medium was changed to serum-free medium for another 12h starvation. The inhibitor was added to 96-well plates for 1h before cells were stimulated by various concentrations of Ang. After incubation for 48 h, cell growth was measured by MTT proliferation assay.

### Inhibition of Ang nuclear translocation by Neomycin

Cells were treated by the procedures described in “Inhibition of NF-κB signaling pathway”，IKK inhibitor was used instead of neomycin (Solarbio, Beijing, China) and the nuclear protein was extracted.

### Statistics

All data were expressed as mean±SD, using the Student *t*-test and one-way ANOVA methods for statistical analysis by SPSS13.0 and MxPro3000-QPCR statistical softwares, respectively. *p<0.05 means was used to indicate the statistically significant difference.

## Results

### Analysis of Ang expression in normal brain and astrocytoma tissues

**Fig 1:**
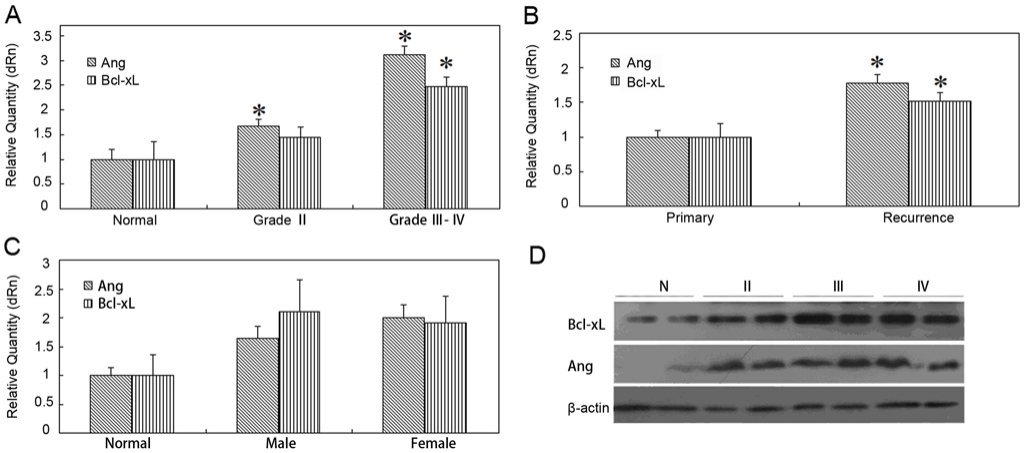
Analysis of Ang and Bcl-xL expression in normal brain tissue and various grades of astrocytoma tissues. A~C. The mRNA levels of Ang and Bcl-xL in astrocytoma tissue samples were compared with that in normal brain tissue in terms of grade(A), recurrence(B) and gender(C) respectively (*, p<0.05). D. The Ang and Bcl-xL expression were detected by western blotting assay in tissue samples in terms of grade.

The expression of Ang and Bcl-xL was detected in 34 tissue samples from control people and patients with astrocytoma by RT-qPCR. The results demonstrated that the expression of Ang was significantly increased in the lower (Ⅱ) and higher (Ⅲ, Ⅳ) grades of astrocytomas in comparison with that in the normal brain tissue(p<0.05; p<0.05). The expression of anti-apoptotic protein Bcl-xL also showed a significant increase in the higher grades (Ⅲ, Ⅳ) of astrocytoma (p<0.05) (Fig. 1A). Both Ang and Bcl-xL had a positive correlation with the tumor malignant degree suggesting that Ang may be involved in the regulation of the proliferation and anti-apoptosis of astrocytoma.

Furthermore, the analysis of the above-mentioned clinical cases revealed that the expression level of both Ang and Bcl-xL in the recurrent patients was significantly higher (p<0.05) in comparison with the newly diagnosed patients (Fig. 1B). The disease progress of the recurrent patients would be often significantly faster than that in the non-recurrent patients, so the relationship between the mRNA level of Ang and the tumor recurrence suggested the Ang expression was closely related with the patient’s disease progress. However, the difference at the Ang and Bcl-xL expression level of the astrocytoma samples was not statistically significant between two genders (Fig. 1C).

The protein expression of Ang and Bcl-xL in the patient’s tissue was also higher than that in the normal tissue, which was consistent with the quantitative real-time PCR result (Fig. 1D). As the NF-κB signaling pathway was activated in astrocytoma, Ang may be involved in the disease progression by activating NF-κB pathway and up-regulating the expression of downstream target gene Bcl-xL. In order to further clarify the effect of Ang on astrocytoma and its regulatory mechanism, we selected the polymorphic glioblastoma cell line U87MG for the analysis at cytological level.

### Promotion of U87MG cell proliferation by Ang

**Fig 2:**
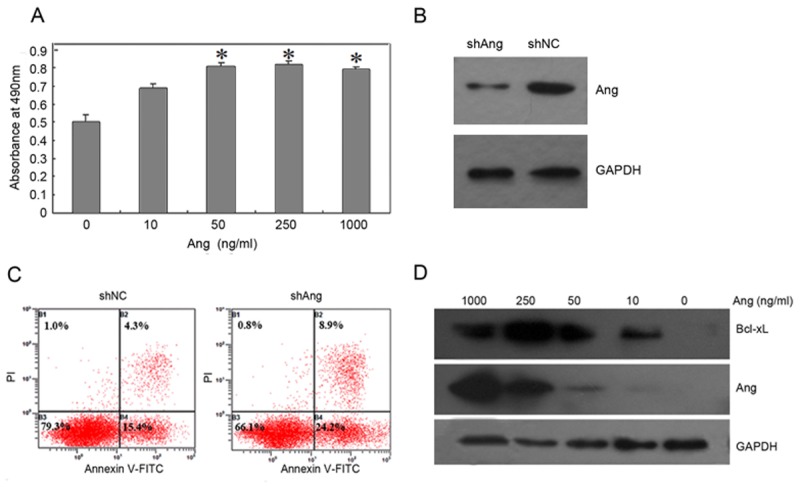
Activity assay of Ang. (A) Indicated amounts of Ang were added to 96-well plates containing 5,000 U87MG cells/well. After incubation for 48h, cell growth was measured by MTT assay. The data represent the means of three independent experiments. B~C. U87MG cells were transfected with shAng or shNC. The cells were lysed 48 h post-transfection and probed with anti-Ang or anti-GAPDH antibodies(B) or analyzed by double staining with Annexin V-FITC/PI and flow cytometry(C). D. The expression of Ang and Bcl-xL were detected in Ang-treated U87MG cells. The cells were incubated with 0, 10, 50, 250, and 1000 ng/ml Ang respectively for 2 h.

The effect of Ang on the U87MG cells was examined by MTT assay. Ang at concentration of 10 ng/ml could stimulate U87MG cell proliferation. The effect of Ang on U87MG cell proliferation displayed a significant increase (p<0.05) from 50 to 1000 ng/ml, and was saturated above 50 ng/ml (Fig. 2A). The result demonstrated that Ang facilitated the U87MG cell proliferation.

ShRNA for Ang was cloned into a eukaryotic expression plasmid pGPU6/GFP/Neo-shRNA for constructing a recombinant plasmid (shAng). After being transfected into U87MG cells for 48h, shAng effectively inhibited the Ang expression in comparison with the control group (shNC) (Fig. 2B). Meanwhile, the cells were stained with Annexin V-FITC/PI double staining method for detection of the apoptosis. The apoptotic rate of U87MG cells was increased by 13.4% after knocking down the expression of Ang in comparison with the control group (Fig. 2C), that was consistent with the effect of Ang-promoting U87MG cell proliferation. Furthermore, Bcl-xL expression was up-regulated with the increase of Ang concentration (Fig. 2D), suggesting that Ang may facilitate cell proliferation by up-regulating Bcl-xL expression.

### Ang promoted cell proliferation by activating NF-κB signaling pathway

**Fig 3:**
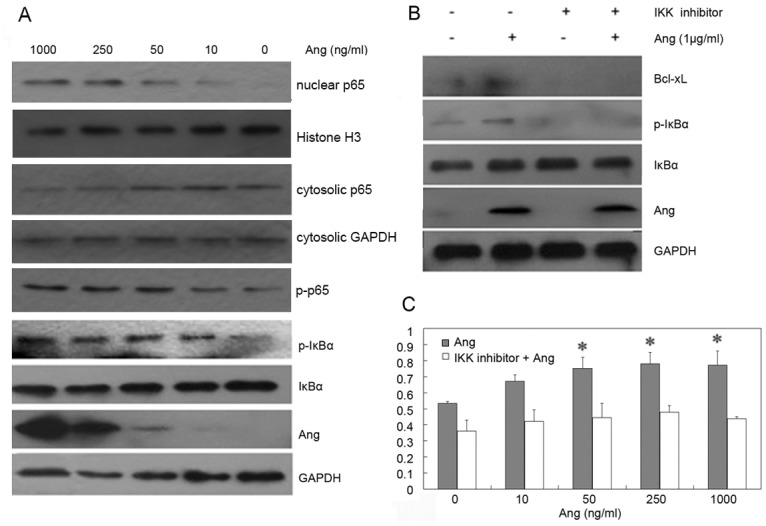
Activation of NF-κB pathway by Ang. A. The cells were incubated with 0, 10, 50, 250, and 1000 ng/ml Ang for 2 h. The proteins of phosphorylated p65, phosphorylated IκBα, IκBα, Bcl-xL, Ang and GAPDH were detected in Ang-treated U87MG cells. The expression levels of p65 and Histone were detected in nuclear extraction preparations. The expression levels of p65 and GAPDH were detected in cytosolic extraction preparations. B. The cells were pretreated by the IKK inhibitor for 1h and were then stimulated by Ang at the final concentration 1μg/ml for 2h. After the protein was extracted, samples with equal amounts of protein were subject to SDS-PAGE and Western blotting analyses for phosphorylation of IκBα, IκBα, Bcl-xL, Ang and GAPDH. C. The inhibitor was added to 96-well plates containing 5,000 U87MG cells/well for 1 h before cells were stimulated by various concentrations of Ang. After a 48 h incubation, cell growth was measured.

To further investigate the relationship between Ang-induced cell proliferation and the activated signaling pathways in GBM, U87MG cells were stimulated with different concentrations of Ang. The results showed that Ang promoted p65 phosphorylation, IκBα phosphorylation and intra-nuclear p65 expression (Fig. 3A). The above-mentioned results confirmed that Ang activated NF-κB signaling pathway in U87MG cell. NF-κB is present in the cytosol in an inactive state, complexed with the inhibitory IκB protein. In the classical pathway, NF-κB is regulated by two kinases, IKKα and IKKβ. The latter is particularly important as it phosphorylates IκB, which is subsequently ubiquitinated and degraded by the 26S proteasome, thus leading to the nuclear translocation of the p50-p65 subunits of NF-κB followed by p65 phosphorylation, acetylation and methylation, DNA binding, and gene transcription [17–18].

IKK inhibitor can inhibit the IKK activity and the activation of its downstream pathway [19]. Our results showed that IKK inhibitor (100 μM) inhibited the phosphorylation of IκBα, Bcl-xL expression and also Ang-induced IκBα phosphorylation and the up-regulation of Bcl-xL expression (Fig. 3B). Then, MTT test also showed that the inhibition of NF-κB pathway suppressed Ang-induced cell proliferation partly (Fig. 3C). The results above indicated that Ang can promote astrocytoma cell proliferation by activating NF-κB signaling pathway.

### The nuclear translocation of Ang is required for Ang-induced cell proliferation

**Fig 4:**
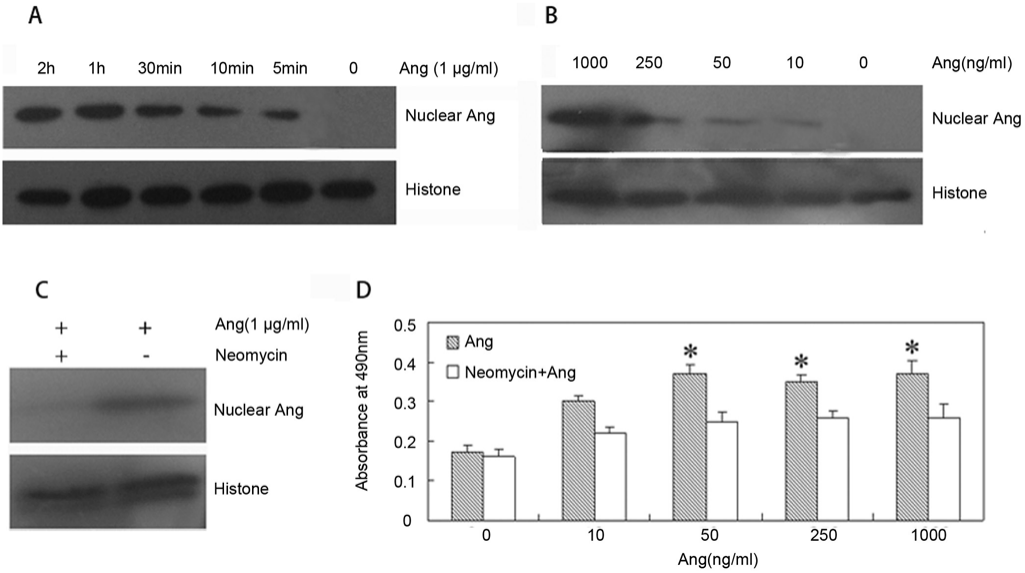
Nuclear function of Ang. A. The cells were incubated with 0, 10, 50, 250, and 1000 ng/ml Ang for 2 h. The protein levels of Ang and Histone were detected in nuclear extraction preparation of Ang-treated U87MG cells. B. The cells were incubated with 1000 ng/ml Ang for 0, 5min, 10min, 30min, 1 h, and 2 h. The expression of Ang and Histone were detected in nuclear proteins of Ang-treated U87MG cells. C. The cells were pretreated with Neomycin for 1h and stimulated by Ang at the final concentration 1μg/ml for 2 h. The protein levels of Ang and Histone were detected in the nuclear extraction. D. Neomycin was added to 96-well plates containing 5,000 U87MG cells/well for 1 h before cells were stimulated by various concentrations of Ang. After a 48 h incubation, cell growth was measured.

In the U87MG cells, the nuclear accumulation of Ang to 1000ng/ml can be readily detected after incubation for 5 minutes (Fig. 4A). Meanwhile, nuclear translocation of exogenous Ang is concentration-dependent. When the concentration of Ang is as low as 10 ng/ml, nuclear accumulation can also be detected (Fig. 4B).

In the human umbilical vein endothelial cells (HUVEc), neomycin inhibited nuclear translocation of Ang and abolished Ang-promoted cell proliferation [20]. In U87MG cell, Neomycin (100 μM) also inhibited nuclear translocation of Ang (Fig. 4C). MTT assay showed that neomycin did not influence the U87MG cell proliferation, but inhibited the Ang-promoted proliferation effect (Fig. 4D). The above-mentioned results demonstrated that Ang promoted the cell proliferation by its nuclear effect on U87MG cell.

### Ang activates NF-κB pathway by regulating the expression of FHL3

**Fig 5:**
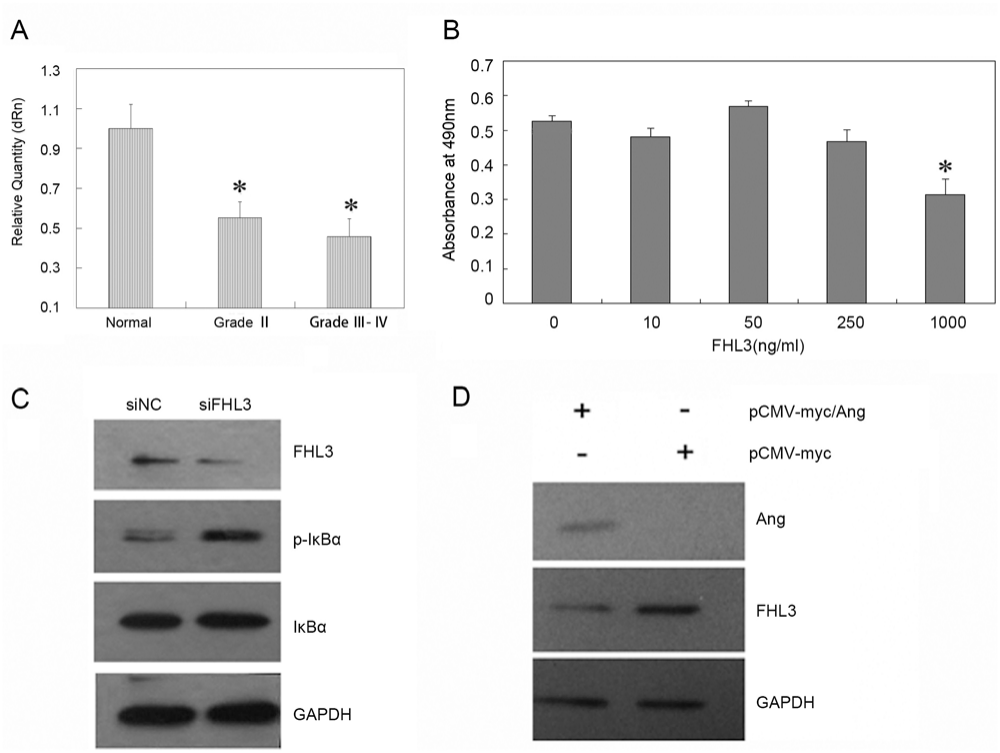
The relationship between FHL3 and Ang. A. The mRNA levels of FHL3 in astrocytoma tissue samples were compared with that in normal brain tissue in terms of grade (*, p<0.05). B. FHL3 at indicated concentrations was added to 96-well plates containing 5,000 U87MG cells/well. After a 48 h incubation, cell growth was measured by MTT assay. The data represents the means of three independent experiments. C. The cells were transfected with siFHL3 or siNC for 48 h. The protein levels of FHL3, phosphorylated IκBα, IκBα and GAPDH were detected in whole cell lysates. D. The cells were transfected with pCMV-myc/Ang or pCMV-myc for 48 h. The protein levels of Ang, FHL3 and GAPDH were detected in whole cell lysates.

Our previous study identified FHL3 as a novel binding partner for Ang. Furthermore, FHL3 is required for Ang-stimulated HeLa cell proliferation and nuclear translocation of Ang. In the current study, we sought to determine whether FHL3 is involved in Ang-regulated glioma progression. First, we assessed the expression of FHL3 in all the tumor and normal tissues. In agreement with previous report, FHL3 expression was decreased in the tumor tissues (Fig. 5A) and FHL3 inhibited U87MG cell proliferation as evaluated by MTT assay (Fig. 5B). In T98G cells (a human glioma cell line), FHL3-induced apoptosis was mediated by caspase3 and Ang took part in the regulation of evolution process of astrocytoma by mediating NF-κB pathway, so we hypothesized that Ang may activate NF-κB pathway by regulating the expression of FHL3 [21]. Downregulation of FHL3 by siRNA promoted IκBα phosphorylation (Fig. 5C) and over-expression of Ang resulted in significantly decreased FHL3 protein levels (Fig. 5D). The results suggested that Ang may promote cell growth by inhibiting FHL3 expression. Based on the previous and present experimental results, FHL3 may play a tumor suppressor—like role in certain cancer cells and the function of Ang appears to be mechanistically linked to the expression of FHL3 protein.

## Discussion

Ang is the only member with angiogenesis ability in the ribonuclease superfamily and angiogenesis is an important step in the process of tumor progression. In addition, Ang expression is up-regulated in numerous tumors and hypoxic circumstances [22], suggesting Ang may be closely related with the tumor genesis. However, the relationship between Ang and the progression of astrocytoma is still unknown. Astrocytoma is the most common subtype of human brain glioma which results from malignant change of astrocyte and can be classified into 4 grades according to WHO classification criteria 2000: pilocytic astrocytoma (grade I), diffuse astrocytoma (grade Ⅱ), anaplastic astrocytoma (grade Ⅲ) and glioblastoma (GBM, grade Ⅳ) [23]. Average survival time of the patients with astrocytoma is about 2~4 years after surgery, while that of GBM patients is less than one year. In regard with cell necrosis and endothelial proliferation, GBM is significantly different from the lower grades of astrocytoma. GBM can be either primary or derived from malignization of the lower grades of astrocytoma. During this process, the related signal pathways within tumor cells are also changed. Thus even the multi-modal therapeutic procedures have been improved continuously, including surgery, radiotherapy and chemotherapy, there have been no way to extend the survival time of GBM patients.

By means of detection and analysis of clinical brain glioma specimen, we found that Ang plays an important role in the genesis and development of brain glioma, and was related closely with the disease progression in patients. Firstly, we found that Ang expression positively correlated to the malignant degree of astrocytoma and recurrent patients. *In vitro*, Ang promoted U87MG cell proliferation and the down-regulation of Ang expression induced cell apoptosis. Besides, Ang up-regulated the expression of anti-apoptotic protein Bcl-xL in U87MG cells. Because Ang displayed anti-apoptosis activity by Bcl family proteins and NF-κB pathway in pluripotential p19 mouse carcinoembryonic cells, we considered that the anti-apoptotic activity of Ang in astrocytoma may be accomplished possibly by regulating the expression of Bcl-xL and NF-κB pathway.

Currently the exact pathogenesis of brain glioma is not yet clear, a widespread viewpoint is that the genesis of brain glioma correlated with activation of the serial signaling pathways resulting in disordered or excessive cell growth [24]. ERK1/2 was extensively activated in the brain glioma tissues [25–26], suggesting the activation of ERK1/2 pathway was related with the genesis of brain glioma. NF-κB pathway was considered as the treatment target and was activated in GBM, blocking of which promoted the senescence of differential cells. Although Ang took part in the regulation of MAPK and NF-κB signaling pathways, our results indicated that Ang did not promote ERK1/2 phosphorylation in U87MG cell (data not shown), but promoted the U87MG cell proliferation by inducing the activation of NF-κB pathway. Therefore, NF-κB pathway in astrocytoma is one of the important pathways by which Ang regulates the tumor genesis and development.

However, whether Ang participates extensively in the regulation of intracellular signaling pathways has been unknown because of the still ongoing exploration of Ang receptor. The conceivable action mechanism of Ang is mainly as follows: a dissociable smooth muscle-type α-actin, Ang binding protein located on the cell surface, is involved in Ang-induced angiogenesis by stimulating cell-associated proteolytic activities and endothelial cell invasion [27]. When cells are cultured under sparse density, they express a 170 kDa putative cell surface receptor that is as equally necessary as the binding protein [28]. After entering the cells via this putative receptor, Ang acts at the cellular level by stimulating cell signaling pathways and undergoing nuclear translocation. On one hand, Ang activates ERK, SAPK/JNK, and NF-κb pathway in different cells. On the other hand, Ang translocates to nucleus rapidly and the internalized Ang is eventually accumulated in the nuclei [29]. Nuclear translocation of Ang is a critical step in the process of angiogenesis and cancer cell proliferation. In U87MG cells, Ang also enter cell nucleus. Neomycin was used to inhibit Ang from entering nucleus and also partially suppressed Ang-induced cell proliferation, which is consistent with the intranuclear effect of Ang in HUVEc. The above-mentioned results further suggested that Ang promotes U87MG cell proliferation by mediating distinct signaling pathways and its nucleus function.

To better clarify the association between Ang expression and evolution process of astrocytoma, the interaction between Ang and FHL3 was confirmed with GST pull-down and co-IP assays in our previous study. FHL3 is a member of LIM protein superfamily, which interacts with proteins through its LIM domains and inhibits tumor cell proliferation by Smads-mediated signaling pathway [30]. Moreover, FHL3 interacts with ERK2 and plays an important role in regulating ERK pathway [31]. In our study, the expression of FHL3 negatively correlated to the malignant grades of astrocytomas. FHL3 in high concentration inhibited U87MG cell proliferation, and phosphorylated IκBα was increased in FHL3-knocked down cells. Over-expression of Ang inhibited the expression FHL3 protein. These results suggest that Ang may activate NF-κB pathway by regulating the expression of FHL3. Therefore, clarifying the molecular regulation mechanism of Ang in glioblastoma provides not only new theories and clues to explore the anti-apoptosis mechanism of Ang, but also a new strategy for clinical therapy against astrocytoma, identifying a new target for the development of anti-cancer new drugs.
